# Higher Donor Age and Severe Microvascular Inflammation Are Risk Factors for Chronic Rejection After Treatment of Active Antibody-Mediated Rejection

**DOI:** 10.3389/ti.2024.11960

**Published:** 2024-02-02

**Authors:** Taro Banno, Toshihito Hirai, Rikako Oki, Takafumi Yagisawa, Kohei Unagami, Taichi Kanzawa, Kazuya Omoto, Tomokazu Shimizu, Hideki Ishida, Toshio Takagi

**Affiliations:** ^1^ Department of Urology, Tokyo Women’s Medical University Hospital, Tokyo, Japan; ^2^ Department of Organ Transplant Medicine, Tokyo Women’s Medical University Hospital, Tokyo, Japan

**Keywords:** antibody-mediated rejection, Banff classification, graft survival, kidney transplantation, treatment outcomes

## Abstract

Recent developments in intensive desensitization protocols have enabled kidney transplantation in human leukocyte antigen (HLA)-sensitized recipients. However, cases of active antibody-mediated rejection (AABMR), when they occur, are difficult to manage, graft failure being the worst-case scenario. We aimed to assess the impact of our desensitization and AABMR treatment regimen and identify risk factors for disease progression. Among 849 patients who underwent living-donor kidney transplantation between 2014 and 2021 at our institution, 59 were diagnosed with AABMR within 1 year after transplantation. All patients received combination therapy consisting of steroid pulse therapy, intravenous immunoglobulin, rituximab, and plasmapheresis. Multivariable analysis revealed unrelated donors and preformed donor-specific antibodies as independent risk factors for AABMR. Five-year death-censored graft survival rate was not significantly different between patients with and without AABMR although 27 of 59 patients with AABMR developed chronic AABMR (CABMR) during the study period. Multivariate Cox proportional hazard regression analysis revealed that a donor age greater than 59 years and microvascular inflammation (MVI) score (g + ptc) ≥4 at AABMR diagnosis were independent risk factors for CABMR. Our combination therapy ameliorated AABMR; however, further treatment options should be considered to prevent CABMR, especially in patients with old donors and severe MVI.

## Graphical Abstract

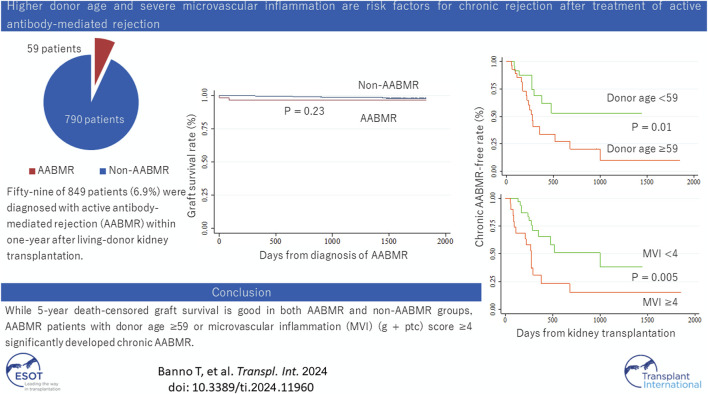

## Introduction

Long-term graft survival has steadily improved over the past decades owing to advances in the care of transplant recipients [1]. Acute allograft rejection rates have also steadily decreased due to the use of immunosuppressive regimens targeting early rejection. In the current era, the incidence of acute rejection has decreased from rates exceeding 50% during the 1970s to between 10% and 20%. However, the situation is entirely different for patients who have sensitization against human leukocyte antigens (HLA).

Moreover, the number of HLA incompatibility adversely affects graft outcomes although the introduction of modern immunosuppression has lessened the degree of this impact over time [2]. AABMR, which is associated with HLA mismatch, HLA incompatibility, and blood group incompatibility, is an independent risk factor for death-censored graft failure [3]. Moreover, graft survival is significantly worse, especially from chronic allograft nephropathy, in those with AABMR than in those with acute rejection without evidence of AABMR [4]. Despite the development of immunosuppressive therapies over the decades, AABMR remains a cause of declining long-term graft survival.

Although several studies on treatments for AABMR have been reported, including plasmapheresis, IVIG, steroid pulse therapy, and anti-CD20 monoclonal antibody (rituximab) administration [5–12], a consensus on the therapeutic strategy for AABMR remains elusive. Furthermore, chronic AABMR (CABMR), characterized by transplant glomerulopathy (the result of remodeling glomeruli and microvascular injury), is unlikely to be reversed by current therapies [13].

In Japan, intravenous immunoglobulin (IVIG) was approved as a desensitization regimen in 2019 and is now covered by public health insurance. Thereafter, the number of kidney transplantation cases in highly HLA-sensitized recipients increased. Therefore, we are concerned that the number of cases of severe AABMR has increased and resulted in poorer graft outcomes. Although it had not been covered by the insurance, we have used high-dose IVIG for the desensitization and the AABMR treatment since before 2019. We conducted this study to evaluate the treatment outcomes, including impact on the Banff score, and identify risk factors for CABMR development in patients with living-donor kidney transplantation, including HLA-incompatible recipients.

## Materials and Methods

### Ethics Statements

This study was approved by the Health Sciences Institutional Review Board (IRB) of Tokyo Women’s Medical University Hospital (approval number: 4460-R), and the procedures followed were in accordance with the ethical standards of the local IRB and with the Helsinki Declaration of 1975, as revised in 2013. Informed consent was waived because patient data were extracted as anonymized data.

### Study Design and Participants

This single-center retrospective study included a recent patient cohort including HLA-sensitized recipients. Between 2014 and 2021, 894 kidney transplantations were performed at Tokyo Women’s Medical University Hospital, including 849 living-donor and 45 deceased-donor transplantations. Protocol allograft biopsies were routinely performed 3 months and 1 year after kidney transplantation. For-cause allograft biopsies were also performed in patients with delayed graft function, serum creatinine level elevation, increased proteinuria, and *de novo* donor-specific antibody (DSA) detection. Among the 849 patients with living-donor kidney transplantation, 59 were diagnosed with AABMR within 1 year of kidney transplantation. Follow-up allograft biopsies were conducted approximately 6 months after treatment.

### Patient Monitoring

Data were collected from patients’ medical records. All patients were examined for HLA compatibility with complement-dependent cytotoxicity (CDC) crossmatch (XM), flow cytometry crossmatch (FCXM), or solid-phase immunoassay (SPI) using a single antigen bead assay (LABScreen™ single antigen beads, One Lambda, Canoga Park, CA). Serum creatinine levels, estimated glomerular filtration rate (eGFR), and the presence of proteinuria 6 months after treatment (after) were compared with those at diagnosis (before). eGFR was calculated using revised equations for eGFR from serum creatinine in Japan as follows: eGFR (mL/min/1.73 m^2^) = 194 × serum creatinine (−1.094) × age (−0.287) [×0.739 (if female)] [14]. Anti-HLA antibodies were screened using LABScreen™ single antigen beads 1 year after transplantation or when ABMR was suspected. We defined positive DSA when an anti-HLA antibody to the donor was detected by SPI.

### Transplant Biopsy and Pathological Diagnosis

Renal allograft biopsies were performed using an ultrasound-guided percutaneous technique, and two cores were collected per biopsy using a 16-gauge needle. Histomorphology was evaluated in formalin-fixed paraffin-embedded sections using a standard methodology. Pathological diagnosis was retrospectively reviewed and uniformed according to the Banff criteria 2019 as stated below [15].

#### Active ABMR

1. Histological evidence of acute tissue injury, which may include one or more of the following:
  • Microvascular inflammation (MVI) (g > 0 and/or ptc >0), in the absence of recurrent or *de novo* glomerulonephritis, although in the presence of acute T-cell mediated rejection (TCMR), borderline infiltrate, or infection, ptc ≥1 alone is not sufficient and g must be ≥1
  • Intimal or transmural arteritis.
  • Acute thrombotic microangiopathy, in the absence of any other cause.
  • Acute tubular injury, in the absence of any other apparent cause.
2. Evidence of current/recent antibody interaction with vascular endothelium, including one or more of the following:
  • Linear C4d staining in peritubular capillaries or medullary vasa recta.
  • At least moderate MVI ([g + ptc] ≥ 2) in the absence of recurrent or *de novo* glomerulonephritis, although in the presence of acute TCMR, borderline infiltrate, or infection, ptc ≥2 alone is not sufficient, and g must be ≥1.
  • Increased expression of gene transcripts/classifiers in the biopsy tissue is strongly associated with ABMR if thoroughly validated.
3. Serologic evidence of circulating DSA. C4d staining substitutes for DSA in cases without DSA. Patients negative for both DSA and C4d were classified into suspected AABMR. Non-HLA antibodies were not routinely examined in the current study.

#### Chronic Active ABMR

1. Morphologic evidence of chronic tissue injury, including one or more of the following:
  • Transplant glomerulopathy (cg > 0) if there is no evidence of chronic thrombotic microangiopathy or chronic recurrent/*de novo* glomerulonephritis, including changes evident by electron microscopy alone.
  • Severe peritubular capillary basement membrane multilayering.
  • Arterial intimal fibrosis of new onset, excluding other causes.
2. Identical to criterion 2 for active ABMR, as stated above.
3. Identical to criterion 3 for active ABMR, as stated above, including a strong recommendation for DSA testing whenever criteria 1 and 2 are met.

### Immunosuppressive Regimen and Desensitization

Patients undergoing kidney transplantation at our institution started a triple immunosuppressive regimen including a calcineurin inhibitor (tacrolimus), an anti-proliferative agent (mycophenolate mofetil), and steroid (methylprednisolone) 7 days before transplantation as immunosuppression induction. Furthermore, the non-depleting anti-CD25 monoclonal antibody (basiliximab) was routinely induced twice: on the day of transplantation and postoperative day 4. ABO blood type-incompatible patients received additional desensitization with rituximab (200 mg/body) and plasmapheresis (2–4 times) until the anti-blood type IgG and IgM antibody titers decreased to <1:32, according to our protocol as we have reported before [16, 17]. Regarding HLA-incompatible kidney transplantation, the desensitization in patients with mean fluorescence intensity (MFI) of DSA <3,000 and negative for CDC and FCXM was performed according to ABO blood type-incompatible kidney transplantation. High-dose IVIG (2 g/kg) is added to HLA-incompatible patients with MFI of DSA ≥3,000 or positive for CDC or FCXM, in addition to ABO blood type-incompatible kidney transplantation protocol [18–20]. Maintenance immunosuppression included tacrolimus (trough value of approximately 5 ng/mL), mycophenolate mofetil acid (500–750 mg), and methylprednisolone (2–4 mg).

### Treatments for Active Antibody-Mediated Rejection

All the patients with AABMR were treated with methylprednisolone administration at 500 mg for two consecutive days, except patients with subclinical AABMR with diabetes or other complications. Patients diagnosed with for-cause biopsy (clinical AABMR) or those with protocol biopsy (subclinical AABMR) with eGFR <25 mL/min, MVI (g + ptc) score ≥4, or positive for *de novo* DSA were considered for IVIG administration/plasma pheresis, which has been known to improve graft survival [21–24], when patients agreed after giving informed consent. Rituximab administration was considered when CD19+B cells remained detectable.

### Statistical Analysis

Continuous variables are expressed as mean and standard deviation or median and interquartile range (IQR), while categorical variables are expressed as percentages. Independent continuous variables were analyzed using the *t*-test for normally distributed data and the Wilcoxon rank-sum test for non-normally distributed data, and categorical variables were analyzed using the Pearson χ-square test. Paired t-tests and Wilcoxon signed-rank tests were used to analyze dependent continuous variables. McNemer’s test was used for dependent categorical variables. Univariable and multivariable Cox proportional hazard regression models were used to assess the hazard risk. Continuous variables were converted into categorical variables in Cox proportional hazard regression analysis. Kaplan-Meier curves and Log-rank tests were generated to compare the time until an event occurs between the different groups. Statistical significance was set at *p* < 0.05. Analyses were performed using Stata, version 15.1 (Stata Corp. LP, College Station, TX, United States).

## Results

### Patient Background Characteristics

Table 1 presents the patient background characteristics. Fifty-nine of 849 patients with living-donor kidney transplantations (6.9%) developed AABMR or suspected AABMR (AABMR group) within 1 year of kidney transplantation. The recipient age and rate of unrelated donors were significantly higher in the AABMR group than in the non-AABMR group. Patients in the AABMR group showed a higher immunological risk compared to those in the non-AABMR group (higher rate of history of kidney transplantation, positivity for CDC-XM, FCXM, and SPI). The patients with ABO incompatibility showed a trend of higher frequency in the AABMR group though the difference did not reach statistical significance (*p* = 0.051). AABMR was diagnosed 90 days (IQR: 3–105) after kidney transplantation. Twenty-seven patients (45.8%) with AABMR were diagnosed by for-cause biopsy findings and the remaining 32 (54.2%) were diagnosed by protocol biopsy results. Out of 59 AABMR patients, 36 (61.0%) had preformed DSA, with 8 in class 1, 19 in class 2, and 9 in both classes. Seventeen (28.8%) of 59 in the AABMR group had *de novo* DSA, with 2 in class 1 and 15 in class 2. The immunodominant MFI values were 1900 (1,247–10,843) for preformed DSA and 3,181 (1,541–4,713) for *de novo* DSA. Of the 59 patients, 16 (27.1%) did not show either preformed or *de novo* DSAs. However, eight patients were positive for C4d staining, which could substitute for DSA as per the 2019 Banff criteria. Among the remaining eight patients who were negative for both DSA and C4d staining and had MVI scores all ≥2 (suspected AABMR), the rate of developing CABMR was similar to that of patients positive for either DSA or C4d with MVI score ≥2, as shown in Supplementary Figure S1 (*p* = 0.41).

**TABLE 1:** Patient characteristics with living-donor kidney transplantation.

	Total	AABMR^1^	Non-AABMR	*p*-value
*N*	849	59	790	
Recipient age at transplantation (years), mean (SD^2^)	49.1 (13.3)	55.1 (9.3)	48.6 (13.5)	<0.001
Donor age at transplantation (years), mean (SD)	59.6 (10.1)	57.4 (8.3)	59.8 (10.2)	0.08
Recipient sex				
Male, *n* (%)	558 (65.7)	33 (55.9)	525 (66.5)	0.10
Female, *n* (%)	291 (34.3)	26 (44.1)	265 (33.5)	
Donor sex				
Male, *n* (%)	289 (34.0)	25 (42.4)	264 (33.4)	0.16
Female, *n* (%)	560 (66.0)	28 (53.8)	532 (66.7)	
Relation of donor				
Relative, *n* (%)	450 (53.0)	11 (18.6)	439 (55.6)	<0.001
Unrelated, *n* (%)	399 (47.0)	48 (81.4)	351 (44.4)	
ABO-incompatible transplantation, *n* (%)	250 (29.5)	24 (40.7)	226 (28.6)	0.051
Number of kidney transplantations				
Primary, *n* (%)	770 (90.7)	48 (81.4)	722 (91.4)	0.01
Multiple, *n* (%)	79 (9.3)	11 (18.6)	68 (8.6)	
Complement-dependent cytotoxicity				
T-cell positive, *n* (%)	0 of 845 (0)	0 of 59 (0)	0 of 786 (0)	
B-cell positive, *n* (%)	8 of 845 (0.9)	3 of 59 (5.1)	5 of 786 (0.6)	0.001
Flow cytometry crossmatch				
T-cell positive, *n* (%)	35 of 843 (4.2)	14 of 59 (23.7)	21 of 784 (2.7)	<0.001
B-cell positive, *n* (%)	13 of 843 (1.5)	6 of 59 (10.2)	7 of 784 (0.9)	<0.001
Presence of preformed DSA^3^, *n* (%)	131 of 833 (15.7)	35 of 59 (59.3)	96 of 774 (12.4)	<0.001
Allograft weight (grams), mean (SD)	176.9 (44.6)	188.7 (48.2)	176.0 (44.3)	0.04
Warm ischemia time (minutes), mean (SD)	3.6 (1.1)	3.5 (0.9)	3.6 (1.2)	0.37
Total ischemia time (minutes), mean (SD)	72.9 (25.4)	77.6 (24.1)	72.5 (25.5)	0.14
Follow-up period (days), median (IQR^4^)	1,544 (903–2,356)	1,365 (714–2,237)	1,549 (917–2,363)	0.15

AABMR^1^, active antibody-mediated rejection; SD^2^, standard deviation; DSA^3^, donor-specific antibody; IQR^4^, interquartile range.

### Risk Factors for Active Antibody-Mediated Rejection Within 1 year of Kidney Transplantation

**FIGURE 1:**
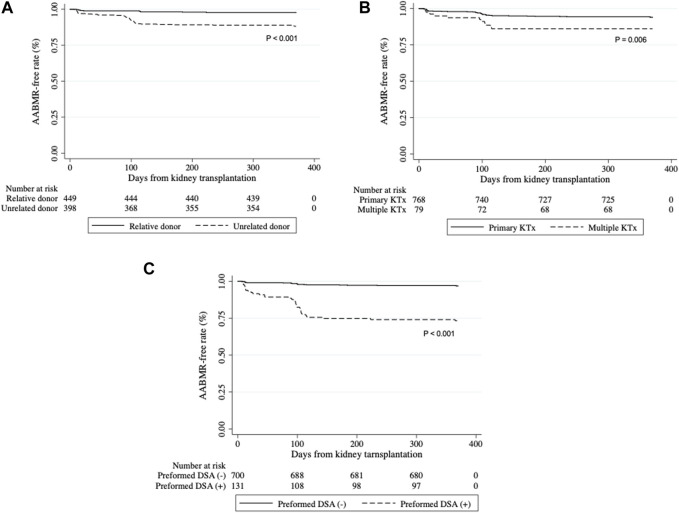
Development of active antibody-mediated rejection (AABMR) within 1 year after kidney transplantation Kaplan-Meier curves for AABMR-free survival between **(A)** relative and unrelated donors, **(B)** primary and multiple kidney transplantation (KTx), and **(C)** positive and negative for preformed donor-specific antibody (DSA). *p*-values calculated by Log-rank tests were shown.

Univariable and multivariable Cox proportional hazard regression analyses were conducted to assess the hazard risk of AABMR over time after kidney transplantation within 1 year of kidney transplantation. In the univariable analysis, variables including age and sex of the recipient and donor; the relationship between the donor and recipient; ABO blood type compatibility and history of previous kidney transplantation; results of CDC-XM, FCXM, and SPI; allograft weight; and warm and total ischemia times were considered as covariables (Table 2). Recipient age greater than 50 years (hazard ratio [HR]: 2.72, 95% CI: 1.51–4.90, *p* = 0.001), unrelated donor (HR: 5.58, 95% CI: 2.82–11.0, *p* < 0.001), history of previous kidney transplantation (HR: 2.44, 95% CI: 1.26–4.70, *p* = 0.008), positive CDC-XM for B cells (HR: 6.84, 95% CI: 2.14–21.9, *p* = 0.001), positive FCXM (T cells, HR: 9.25, 95% CI: 5.05–16.9, *p* < 0.001; B cells, HR: 8.17, 95% CI: 3.26–20.5, *p* < 0.001), and positive SPI (HR: 9.72, 95% CI: 5.70–16.6, *p* < 0.001) were significantly associated with the incidence of AABMR within 1 year after kidney transplantation. The multivariable analysis was performed with selected variables that were statistically significant in the univariable analysis. We chose SPI for immunological risk because CDC, FCXM, and SPI tests may cause multicollinearity. Finally, unrelated donor (HR: 4.48, 95% CI: 2.05–9.79, *p* < 0.001), multiple transplantation (HR: 1.99, 95% CI: 1.01–3.91, *p* = 0.05), and positive SPI (HR: 7.05, 95% CI: 4.16–12.4, *p* < 0.001) were associated with an increased hazard risk of AABMR over time. Significant differences were shown in Kaplan-Meier curves depicting the free rate of AABMR comparing unrelated and relative donors, primary and multiple kidney transplantations, and positive and negative for SPI (Figures 1A–C). Conversely, ABO compatibility was not significantly different (Supplementary Figure S2; *p* = 0.11) although there was a nearly significant difference in the chi-square test.

**TABLE 2:** Cox proportional hazard regression analysis of variables associated with active antibody-mediated rejection within 1 year after kidney transplantation.

		Univariate				Multivariate			
		HR^1^	95% CI^2^		*p*-value	HR	95% CI		*p*-value
Recipient age	<50 years	reference				reference			
	≥50 years	2.72	1.51	4.90	0.001	1.04	0.53	2.05	0.90
Donor age	<60 years	reference							
	≥60 years	0.77	0.46	1.30	0.34				
Recipient sex	Female	reference							
	Male	0.66	0.39	1.11	0.12				
Donor sex	Female	reference							
	Male	1.54	0.91	2.59	0.12				
Relation of donor	Relative	reference				reference			
	Unrelated	5.58	2.82	11.0	<0.001	4.48	2.05	9.79	<0.001
ABO compatibility	Compatible	reference							
	Incompatible	1.53	0.90	2.61	0.12				
Number of transplantations	Primary	reference				reference			
	Multiple	2.44	1.26	4.70	0.008	1.99	1.01	3.91	0.05
CDC^3^ for B cells	Negative	reference							
	Positive	6.84	2.14	21.9	0.001				
FCXM^4^ for T cells	Negative	reference							
	Positive	9.25	5.05	16.9	<0.001				
FCXM for B cells	Negative	reference							
	Positive	8.17	3.26	20.5	<0.001				
Solid–phase immunoassay	Negative	reference				reference			
	Positive	9.72	5.70	16.6	<0.001	7.05	4.16	12.4	<0.001

HR^1^, hazard ratio; CI^2^, confidence interval; CDC^3^, complement-dependent cytotoxity; FCXM^4^, flow cytometry crossmatch.

### Long-Term Outcomes

**FIGURE 2:**
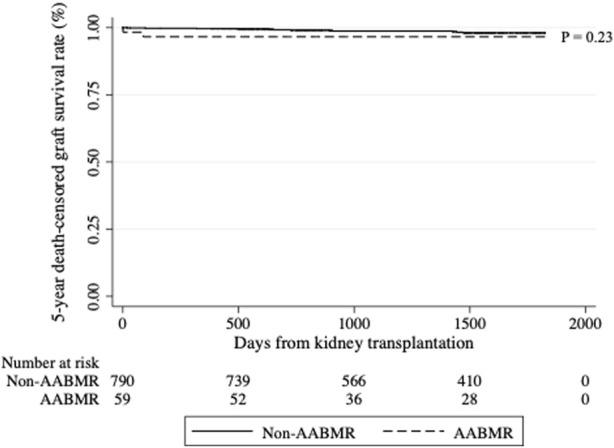
Five-year death-censored graft survival rate between active antibody-mediated rejection (AABMR) and non-AABMR groups. The graft survival rates were compared between patients with AABMR within 1 year of kidney transplantation and those without it. The five-year death-censored graft survival rates did not differ between the groups. Kaplan-Meier curves depicting the five-year death-censored graft survival rate from kidney transplantation were presented, and the *p*-value was calculated using the Log-rank test.

Figure 2 shows a comparison of the Kaplan-Meier curves of five-year death-censored graft survival between the AABMR and non-AABMR groups. Five-year death-censored graft survival rates were 96.7% and 98.0% in the AABMR and non-AABMR groups, respectively (*p* = 0.23). Collectively, these data suggest that most patients sustain long-term renal function after overcoming AABMR.

### Treatments for AABMR

**FIGURE 3:**
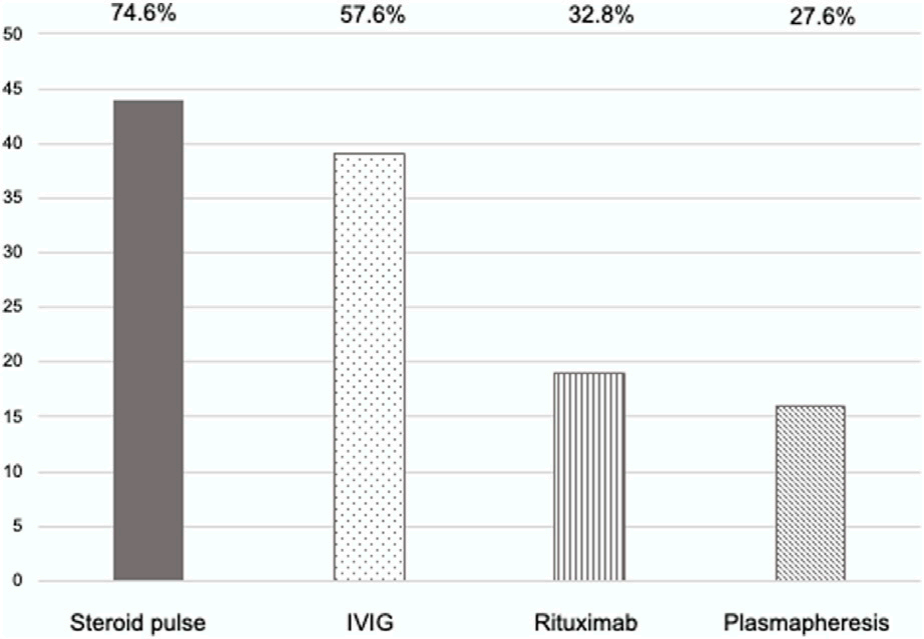
The treatment regimen for active antibody-mediated rejection. *Y*-axis: number of patients. Steroid pulse therapy, methylprednisolone, 250–2,500 mg/body; intravenous immunoglobulin (IVIG) therapy, 0.5–5.2 g/kg; rituximab administration, 200–300 mg/body; plasmapheresis, 2–10 times including plasma exchange or double-filtration plasmapheresis with fresh frozen plasma or albumin replacement.

As shown in Figure 3, patients with AABMR underwent comprehensive anti-humoral immunity treatments. IVIG administration was undertaken for 74% of the patients who met the treatment criteria (18 out of 21 patients [86%] with clinical AABMR and 10 out of 17 [59%] with subclinical AABMR). Rituximab and plasmapheresis were undertaken for 32.8% and 27.6% of the AABMR patients, respectively. The dose of basic immunosuppressants was also adjusted according to the patient’s physical condition.

### Treatment Effects for AABMR

We compared the serum creatinine levels, eGFR, proteinuria, and Banff scores before and after the treatment in 59 patients with AABMR (Table 3). Although two of the 59 patients with AABMR showed an immediate decrease in the eGFR and lost their graft due to hyper-AABMR that did not respond to any treatment, the serum creatinine level and eGFR were significantly improved from 1.8 ± 1.6 mg/dL and 40.0 ± 16.5 mL/min/1.73 m^2^ to 1.4 ± 1.0 mg/dL and 43.7 ± 13.1 mL/min/1.73 m^2^, respectively, after the treatment (*p* = 0.001 and 0.009, respectively). The value of proteinuria was also statistically improved from none (54.2%), 1+ (30.5%), 2+ (13.6%), and 3+ (1.7%) to none (77.8%), 1+ (18.6%), 2+ (3.4%), and 3+ (0%) (*p* = 0.003). Regarding Banff scores, 12 out of 59 patients had a g score of three, and 3 out of 59 patients had a ptc score of three at diagnosis of AABMR, respectively. Fifty-three of the 59 patients underwent a follow-up biopsy after treatment, with a median time of 219 days (IQR: 112–280) after AABMR diagnosis. The proportion of cases with severe g and ptc scores (≥2) significantly reduced at the follow-up biopsy (from 47.2% to 33.9%, *p* = 0.01; from 52.8% to 32.1%, *p* = 0.03, respectively), whereas no alternation was observed in scores for i, t, and C4d. In contrast, the proportion of severe cv scores (≥2) significantly increased in the follow-up biopsy (from 0% to 7.6%, *p* = 0.04).

**TABLE 3:** Renal function, presence of proteinuria, and Banff classification scores at diagnosis of active antibody-mediated rejection and follow-up allograft biopsy.

	At diagnosis with AABMR^1^	At follow-up	*p*-value
Serum creatinine, mean (SD^2^)	1.8 (1.6)	1.4 (1.0)	0.001
eGFR^3^ (mL/min/1.73 m^2^), mean (SD)	40.0 (16.5)	43.7 (13.1)	0.009
Proteinuria, *n* (%)			
None	32 (54.2)	46 (77.8)	0.003
1+	18 (30.5)	11 (18.6)	
2+	8 (13.6)	2 (3.4)	
3+	1 (1.7)	0 (0)	
Banff classification score			
i score ≥2, *n* (%)	1 of 53 (1.9)	2 of 53 (3.8)	0.56
t score ≥2, *n* (%)	2 of 53 (3.8)	3 of 53 (5.7)	0.65
g score ≥2, *n* (%)	25 of 53 (47.2)	20 of 53 (33.9)	0.01
ptc score ≥2, *n* (%)	28 of 53 (52.8)	17 of 53 (32.1)	0.03
C4d score ≥2, *n* (%)	20 of 53 (37.7)	19 of 53 (35.9)	0.76
ci score ≥2, *n* (%)	1 of 53 (1.9)	3 of 53 (5.7)	0.32
ct score ≥2, *n* (%)	1 of 53 (1.9)	3 of 53 (5.7)	0.32
cg score ≥2, *n* (%)	0 of 53 (0)	2 of 53 (3.8)	0.16
cv score ≥2, *n* (%)	0 of 53 (0)	4 of 53 (7.6)	0.04

AABMR^1^, active antibody-mediated rejection; SD^2^, standard deviation; eGFR^3^, estimated glomerular filtration rate.

### Evaluation of Risk for Chronic Active Antibody-Mediated Rejection Development

**FIGURE 4:**
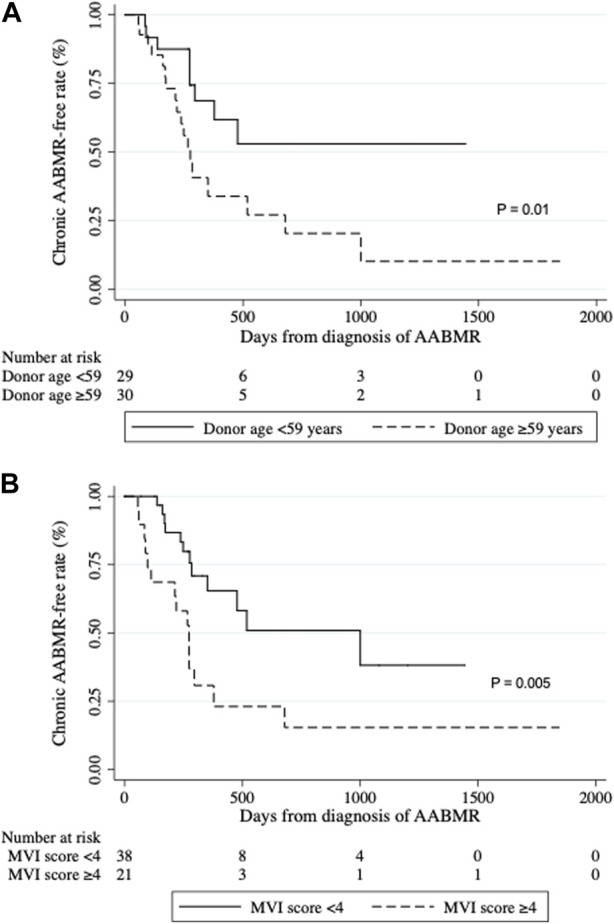
Development of chronic active antibody-mediated rejection (CABMR). Kaplan-Meier curves of the CABMR-free rate after AABMR diagnosis comparing **(A)** the patients with donor ages of ≥59 and <59 years (*p* = 0.01); and **(B)** the patients with MVI score ≥4 and <4 at diagnosis of AABMR (*p* = 0.005). *p*-values calculated by the Log-rank test were shown.

Twenty-seven of 59 patients with AABMR developed CABMR at a median of 248 days (IQR: 137–295) after the initial diagnosis of AABMR. Table 4 shows the results of the univariable and multivariable Cox proportional hazard regression analyses of the variables associated with the development of CABMR in patients with AABMR. Donor age greater than 59 years (HR: 2.68, 95% CI: 1.20–6.01, *p* = 0.02) and MVI (g+ ptc) score ≥4 (HR: 2.85, 95% CI: 1.33–6.10, *p* = 0.01) were significantly associated with the development of CABMR caused by AABMR. Multivariable analysis was conducted using significant variables of univariable analysis. As a result, donor age greater than 59 years (HR: 2.51, 95% CI: 1.12–5.64, *p* = 0.03) and MVI score ≥4 (HR: 2.67, 95% CI: 1.25–5.72, *p* = 0.01) were the independent risk factors for CABMR progression. Kaplan-Meier curves depicting the CABMR-free survival rate revealed significantly poor outcomes after AABMR in cases with older donors and those with severe MVI scores (Figures 4A, B).

**TABLE 4:** Results of Cox hazard regression analysis of variables associated with chronic active antibody-mediated rejection after active antibody-mediated rejection.

		Univariate				Multivariate			
		HR^1^	95% CI^2^		*p*-value	HR	95% CI		*p*-value
Recipient age	<56 years	reference							
	≥56 years	1.72	0.80	3.73	0.17				
Donor age	<59 years	reference				reference			
	≥59 years	2.68	1.20	6.01	0.02	2.51	1.12	5.64	0.03
Diabetes mellitus	Absence	reference							
	Presence	0.96	0.38	2.38	0.93				
ABO compatibility	Compatible	reference							
	Incompatible	0.47	0.19	1.18	0.11				
Number of transplantations	Primary	reference							
	Secondary	2.02	0.80	5.06	0.14				
Number of HLA^3^ mismatches	<4	reference							
	≥4	0.92	0.39	2.19	0.85				
CDC^4^ for B cells	Negative	reference							
	Positive	1.22	0.28	5.25	0.79				
FCXM^5^ for T cells	Negative	reference							
	Positive	1.34	0.58	3.08	0.49				
FCXM for B cells	Negative	reference							
	Positive	2.17	0.63	7.47	0.22				
Solid-phase immunoassay	Negative	reference							
	Positive	1.32	0.56	3.13	0.53				
MFI^6^ of preformed DSA^7^	<5,000	reference							
	≥5,000, <10,000	1.27	0.47	3.45	0.64				
	≥10,000	1.89	0.69	5.17	0.21				
MFI of *de novo* DSA	<3,000	reference							
	≥3,000	0.67	0.20	2.25	0.52				
eGFR^8^ before the treatments	<40 mL/min/1.73 m^2^	reference							
	≥40 mL/min/1.73 m^2^	0.69	0.32	1.47	0.33				
i score at AABMR^9^ diagnosis	<2	reference							
	≥2	1.73	0.23	12.9	0.59				
t score at AABMR diagnosis	<2	reference							
	≥2	3.31	0.77	14.3	0.49				
g score at AABMR diagnosis	<2	reference							
	≥2	1.96	0.91	4.26	0.09				
ptc score at AABMR diagnosis	<2	reference							
	≥2	1.43	0.66	3.09	0.36				
C4d score at AABMR diagnosis	<2	reference							
	≥2	0.97	0.43	2.16	0.94				
MVI^10^ (g+ptc) at AABMR diagnosis	<4	reference				reference			
	≥4	2.85	1.33	6.10	0.007	2.67	1.25	5.72	0.01
Coexistence of TCMR^11^	Absence	reference							
	Presence	0.75	0.10	5.60	0.78				

HR^1^, hazard ratio; CI^2^, confidence interval; HLA^3^, human leucocyte antigen; CDC^4^, complement-dependent cytotoxity; FCXM^5^, flow cytometry crossmatch; MFI^6^, mean fluorescence intensity; DSA^7^, donor-specific antibody; eGFR^8^, estimated glomerular filtration rate; AABMR^9^, active antibody-mediated rejection; MVI^10^, microvascular inflammation; TCMR^11^, T-cell mediated rejection.

## Discussion

We demonstrated favorable treatment outcomes for patients with AABMR within 1 year of kidney transplantation at our institution. The five-year death-censored graft survival rate was 96.2%, and renal function and MVI (g +ptc) were significantly improved after AABMR treatment. Those data collectively suggest the benefit of our new desensitization regimen and AABMR treatment regimen. However, approximately half of the patients with AABMR eventually developed CABMR. We found that older donor age and higher Banff classification g-scores were independent risk factors for the development of CABMR after AABMR diagnosis.

The current study involved patients with high immune risk, whereas the incidence rate of AABMR within 1 year after KTx was not high (6.1%) compared to our previous report, which showed a rate of 10.8% between 2000 and 2008 [25]; this indicates that our current desensitization protocol was successful. Consistent with the results of the current study, a previous study reported that DSAs are a predominant predictor of acute rejection [3]. In contrast, the current study showed that ABO-incompatible kidney transplantation was not related to the development of AABMR. In the reported meta-analysis, including studies from 1999 to 2016, ABO-incompatible transplantation was significantly associated with ABMR compared with ABO-compatible transplantation, and graft survival in ABO-incompatible kidney transplantation was also inferior to ABO-compatible [26]. Indeed, we observed more ABO-incompatible patients in the AABMR group in the current study (Table 1). Conversely, we previously reported that the rate of ABMR and graft survival in ABO-incompatible kidney transplantation was not significantly different from those in ABO-compatible in an era between 2005 and 2013, whereas that was inferior to ABO-compatible between 1989 and 2004 [16]. Consistent with our previous study, Cox proportional hazard regression analysis revealed that the ABO-incompatible transplantation was no longer the risk for AABMR development (Table 2; Supplementary Figure S2). We assume that the development of immunosuppressive agents and the recent desensitization protocol for ABO-blood antibodies decreased the rate of rejection and improved graft survival.

We treated AABMR with combination therapy consisting of steroid pulse therapy, IVIG, rituximab administration, and plasmapheresis. Although two patients had graft loss, most patients showed significant improvements in both renal function and microvascular inflammation. The effective treatments for AABMR were initially thought to be plasmapheresis, which removes humoral mediators from the circulation, and IVIG-inhibiting antibody synthesis [5, 6]. A previous report showed that the combination of plasmapheresis and IVIG significantly improved the one-year graft survival rate compared with plasmapheresis alone [7]. Another study also reported that the combination significantly decreased the graft failure rate (risk ratio: 0.26) compared with a control, with a mean follow-up of 7 years [8]. Furthermore, the addition of rituximab significantly decreased the MFI value of the DSAs and Banff classification scores, resulting in improved graft survival [9–11]. In contrast, a randomized controlled trial did not show a significant difference in one-year graft survival between rituximab and control groups based on plasmapheresis, steroid pulse, and IVIG treatment protocols, whereas microvascular inflammatory scores (glomerulitis and peritubular capillaritis) and chronic injury scores (interstitial fibrosis and tubular atrophy) significantly decreased in the rituximab group [12]. In all studies, the level of evidence for AABMR treatment was low because the data were from a small series. However, the effectiveness of new therapeutic strategies, including proteasome and complement inhibitors, remains unclear [27].

The five-year death-censored graft survival rate of AABMR was 96.2%, which was as good as that in the non-AABMR group (98.5%), indicating the potential of our ABMR treatment regimens. However, 27 of 52 patients with AABMR developed CABMR, which is a well-known risk factor for graft loss [28]. Generally, our treatment regimen effectively prevented early graft loss though it might be difficult to prevent CABMR development and future deterioration of graft function. A longer-term follow-up would be required.

Older donor age was one of the independent risk factors for the development of CABMR. In a previous study, graft survival was lower in transplants from ≥60-year-old donors compared with 18–49-year-old donors. Patient survival was also significantly lower in transplants from donors aged >50 years, compared to transplants from 18 to 49-year-old donors [29]. Similar to our result, a study reported that older donor age was significantly associated with increased susceptibility to chronic allograft damage [30]. Additionally, acute tubular necrosis detected by pretransplant biopsy results or total ischemic time is significantly associated with poor graft outcomes in elderly donors [31, 32]. Irreversible changes may occur if allografts from elderly donors are damaged.

The MVI (g + ptc) score at diagnosis of AABMR was also significantly associated with CABMR development. Several studies have also demonstrated that microvascular injury, including glomerulitis, is correlated with chronic microvascular damage and poor graft prognosis [33–37]. Moreover, graft survival with severe glomerulitis with a g score of three on the Banff classification was 70% a few years after the biopsy [38]. Consistent with these reports, we observed that the MVI score ≥4 was an independent risk factor for CABMR in the current study.

Although CABMR is one of the main causes of late graft failure, there are no approved drugs for its prevention or treatment. A multicenter randomized trial of treatment for transplant glomerulopathy with IVIG and rituximab versus placebo did not show significant differences in eGFR decline, increased proteinuria, Banff classification scores at 1 year, and MFI of immunodominant DSAs [39]. New reagents, such as proteasome inhibitors that eliminate plasma cells producing alloantibodies or anti-C5 monoclonal antibodies that inhibit the activation of C5, did not also show significant improvement in the eGFR and MFI value of DSAs, compared with the control group [40, 41]. More recently, C1 esterase inhibitors that block early complement pathways or inhibitors of the interleukin (IL)-6 and IL-6 receptor axes have been expected to be effective [28].

This study possesses certain limitations. First, it was conducted retrospectively within a singular institution, involving a relatively small cohort. However, the limited number of patients with ABMR is not unexpected, considering the diminishing incidence of acute rejection attributed to advancements in immunosuppressive medications. Second, although the Banff criteria strongly advises testing non-HLA antibodies [42] if HLA antibody testing is negative despite pathological ABMR features, we have not screened out non-HLA antibodies. However, we assume that those cases should be included in AABMR because the rates of development to CABMR following AABMR were similar between true AABMR cases and suspected AABMR cases. Third, because of the retrospective nature of this study, treatments for AABMR were not completely consistent. When adjusting for the severity of ABMR, there was no significant difference in CABMR development between patients treated and those not treated with IVIG (data not shown); however, due to a variety of background differences, we cannot draw the exact conclusion. The clinical impact of IVIG on AABMR needs to be confirmed in future randomized clinical trials.

In conclusion, the AABMR treatment regimen resulted in good short-term graft survival and significant improvements in renal function with reduced Banff scores; however, it did not prevent the development of CABMR. Further treatment options should be considered, especially in patients with older donors and severe MVI.

## Data Availability

The raw data supporting the conclusion of this article will be made available by the authors, without undue reservation.

## Supplementary material

SUPPLEMENTARY FIGURE S1 CABMR free survival rate comparing AABMR and suspected AABMR patients. AABMR, active antibody-mediated rejection; MVI score ≥2 with either DSA- or C4d-positive. suspected AABMR, MVI score ≥2 with both DSA- and C4d-negative. CABMR, chronic active antibody-mediated rejection; DSA, donor-specific antibody. *p* = 0.41 was calculated by the Log-rank test between the groups.

SUPPLEMENTARY FIGURE S2 Development of active antibody-mediated rejection (AABMR) within 1 year after kidney transplantation comparing ABO-compatible and incompatible kidney transplantation. Kaplan-Meier curves for AABMR free survival comparing ABO-compatible and incompatible kidney transplantation. The rates of development of AABMR were similar between both groups (*p* = 0.11).
