# Supplementary figures and images for: Higher Donor Age and Severe Microvascular Inflammation Are Risk Factors for Chronic Rejection After Treatment of Active Antibody-Mediated Rejection
Source: Transpl Int. 2024 Feb 2;37:11960. doi: 10.3389/ti.2024.11960 (PMC10869508; doi:10.3389/ti.2024.11960)

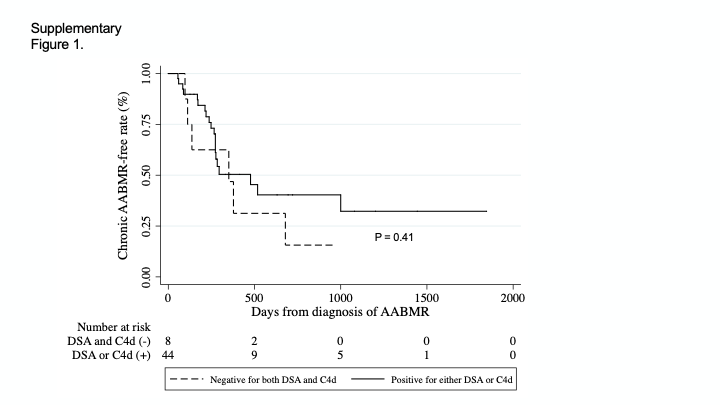

Supplement: Supplementary file 1 [file Image1.TIFF]

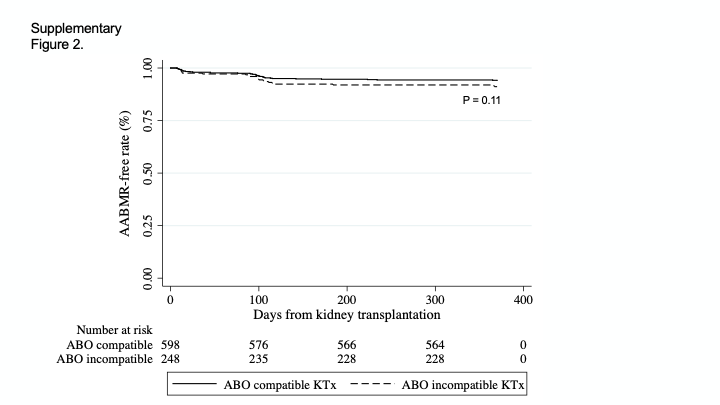

Supplement: Supplementary file 2 [file Image2.TIFF]
